# Inflammatory mediators act at renal pericytes to elicit contraction of vasa recta and reduce pericyte density along the kidney medullary vascular network

**DOI:** 10.3389/fphys.2023.1194803

**Published:** 2023-06-09

**Authors:** Rebecca J. Lilley, Kirsti D. Taylor, Scott S. P. Wildman, Claire M. Peppiatt-Wildman

**Affiliations:** ^1^ Division of Natural Sciences, University of Kent, Kent, United Kingdom; ^2^ Northeastern University London, London, United Kingdom

**Keywords:** inflammation, pericyte, microvasculature, descending vasa recta, microvascular dysregulation

## Abstract

**Introduction:** Regardless of initiating cause, renal injury promotes a potent pro-inflammatory environment in the outer medulla and a concomitant sustained decrease in medullary blood flow (MBF). This decline in MBF is believed to be one of the critical events in the pathogenesis of acute kidney injury (AKI), yet the precise cellular mechanism underlying this are still to be fully elucidated. MBF is regulated by contractile pericyte cells that reside on the descending vasa recta (DVR) capillaries, which are the primary source of blood flow to the medulla.

**Methods:** Using the rat and murine live kidney slice models, we investigated the acute effects of key medullary inflammatory mediators TNF-α, IL-1β, IL-33, IL-18, C3a and C5a on vasa recta pericytes, the effect of AT1-R blocker Losartan on pro-inflammatory mediator activity at vasa recta pericytes, and the effect of 4-hour sustained exposure on immunolabelled NG2+ pericytes.

**Results and discussion:** Exposure of rat and mouse kidney slices to TNF-α, IL-18, IL-33, and C5a demonstrated a real-time pericyte-mediated constriction of DVR. When pro-inflammatory mediators were applied in the presence of Losartan the inflammatory mediator-mediated constriction that had previously been observed was significantly attenuated. When live kidney slices were exposed to inflammatory mediators for 4-h, we noted a significant reduction in the number of NG2+ positive pericytes along vasa recta capillaries in both rat and murine kidney slices. Data collected in this study demonstrate that inflammatory mediators can dysregulate pericytes to constrict DVR diameter and reduce the density of pericytes along vasa recta vessels, further diminishing the regulatory capacity of the capillary network. We postulate that preliminary findings here suggest pericytes play a role in AKI.

## Introduction

Acute kidney injury (AKI) is a global health concern, with ∼13 million cases and ∼1.4 million deaths per year (Ponce and Balbi, 2016). Regardless of the instigating injury, renal diseases have inflammation as a common underlying pathogenic mechanism (Imig and Ryan, 2013). Rapidly post-injury, inflammation is initiated with infiltrating immune cells (Kielar et al., 2005; Basile et al., 2012) and high levels of pro-inflammatory mediators propagating further inflammation and damage. Haemodynamic alterations are also an early notable feature in renal injury (Basile et al., 2012). Importantly, most evidence indicates the renal region primarily affected is the medulla with a disproportionate dysregulation of blood flow (MBF) (Ray et al., 2019; Freitas and Attwell, 2022) (5). In animal models of ischemia reperfusion (IRI), despite post-reperfusion recovery of cortical blood flow, in the medulla a transient improvement of MBF is followed by a progressive gradual decline of up to 50% (Regner and Roman, 2012). Further still, this dysregulation of MBF is thought to be a critical event in the pathogenesis of AKI to CKD, yet the mechanisms behind this remain unclear. Blood flow to the medulla is provided by the vasa recta capillaries (Pallone et al., 2012). Interestingly, the cytokines and complement proteins that infiltrating and residential immune cells secrete, are both directly and indirectly vasoactive (Vila and Salaices, 2005) and involve Angiotensin-II (Ang-II) (Liu et al., 2012). Whilst the inhibition of TNF-α, IL-18, IL-1β and C5a, amongst other inflammatory mediators, has shown to reduce inflammatory injury and preserve renal function (Bonventre and Zuk, 2004; Basile et al., 2012), how these inflammatory mediators may be implicated in the dysregulation of renal blood flow, specifically in the sustained dysregulation of MBF in AKI, is not well characterised.

Our laboratory (Crawford et al., 2011; Crawford et al., 2012), and others (Pallone et al., 2012; Cowley and O’connor, 2013), have demonstrated that the cellular regulators of MBF are resident contractile pericytes, which respond to vasoactive cues from neighbouring endothelial and tubular cells to regulate vasa recta diameter and thus in turn alter flow through these capillaries (Crawford et al., 2011; Cowley and O’connor, 2013). Pericytes have been implicated in progressive chronic kidney disease (CKD), with genetic fate-mapping studies demonstrating pericytes are the major source of myofibroblasts following injury (Lin et al., 2008; Humphreys et al., 2010; Kramann et al., 2015; Lemos et al., 2016). Their subsequent detachment and loss underpins the microvascular rarefaction associated with the progression of AKI to CKD (Lemos et al., 2016; Kramann et al., 2017). Recent work has further highlighted the role of vasa recta pericytes in the medullary no-reflow phenomenon post-reperfusion following renal ischaemia (Freitas and Attwell, 2022), yet the response of pericytes to the early onset renal inflammation that underpins AKI remains less clear. However, the delineation of underlying signalling mechanisms driving this inflammatory response may support the identification of novel targets that are involved in the resultant pericyte-mediated dysregulation of vasa recta diameter, and by extension MBF. As such, the aim of the present study was to use both rat and murine live kidney slice models, in combination with imaging techniques, to investigate the effect of inflammatory mediators on renal pericyte-mediated regulation of vasa recta capillaries.

## Materials and methods

### Tissue slicing

Animal experiments were conducted in accordance with United Kingdom Home Office Scientific Procedures Act (1986). Adult male Sprague-Dawley rats (200–225 g) or adult male C57BL/6J mice (63–70 days; purchased from Charles river UK Ltd., Kent, United Kingdom) were killed by cervical dislocation and kidney tissue slices were obtained as previously described (Crawford et al., 2012). In brief, post removal from the animal kidneys were decapsulated and stored in ice cold physiological saline solution (PSS) bubbled with 95% O_2_/5% CO_2_. Kidneys were sliced using a Leica VT1200S vibrotome tissue slicer (Leica Microsystems Ltd.) PSS contained (mM) 100 NaCl, 5 KCl, 0.24 NaH 2 PO 4, 0.96 Na 2 HPO 4, 10 Na acetate, 1 CaCl 2, 1.2 MgSO 4, 5 glucose, 25 NaHCO 3, 5 Na pyruvate (Sigma-Aldrich Ltd.).

### Live tissue DIC imaging experiments and analysis

**FIGURE 1:**
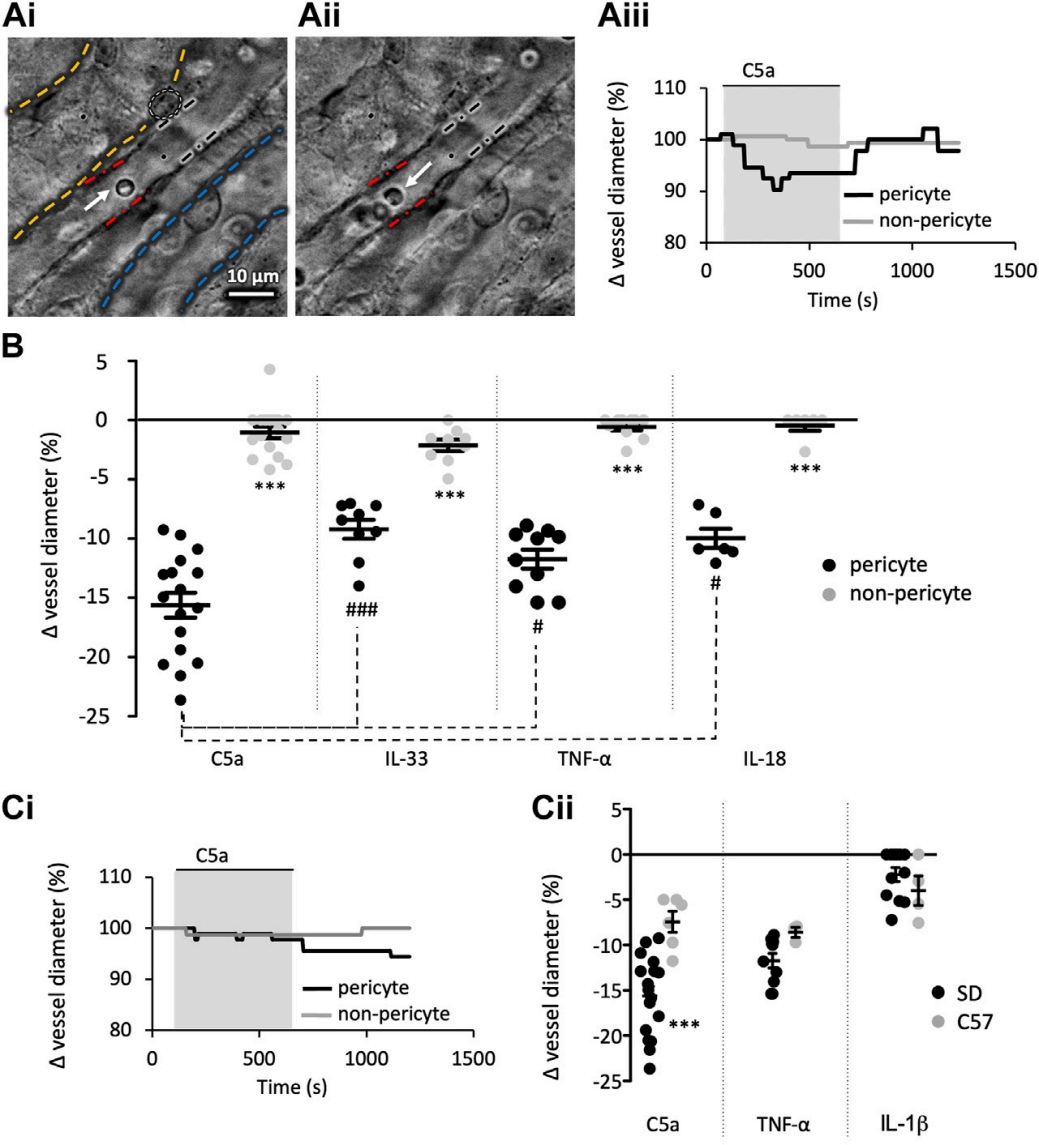
Innate immune components evoke a pericyte mediated constriction of rat and murine vasa recta. Images show a typical field of view of Sprague-Dawley (SD) *in situ* vasa recta during superfusion of tissue with PSS **(Ai)** and agonist **(Aii)**. Pericytes can be seen on capillary walls, denoted by a white oval, and black and red lines indicate areas of pericyte site and non-pericyte measurements respectively. White and blue dashed lines highlight an adjacent collecting duct and tubule respectively. White arrows indicate red blood cells inside the vasa recta. The C57BL/6J (C57) mouse *in situ* vasa recta have a comparable appearance to the rat vasa recta. Scale bar measures 10 μm. Vessel diameter was measured during superfusion of tissue slices with PSS, C5a and a subsequent wash with PSS. The line graph **(Aiii)** shows a representative trace of these measured changes in rat vessel diameter at pericyte (black line) and non-pericyte sites (grey line), showing vessel diameter reduced during exposure of C5a (grey box) at pericyte sites with little change at the non-pericyte site. **(B)** Graph shows percentage change in rat vasa recta diameter at pericyte (black) and non-pericyte sites (grey) in response to C5a, IL-33, TNF-α, and IL-18. TNF-α and C5a were used on C57 tissue as they were more potent components, with IL-1β included as a non-responsive control. **(Ci)** shows a representative murine trace as described in **(Aii)**. **(Cii)** Graph shows percentage change in vasa recta diameter at pericyte sites in SD (black) and the C57 (grey) tissue slices in response to C5a, TNF-α, and IL-1β. Significance was calculated using a nested two-tailed paired Student’s *t*-test for comparisons between pericyte and non-pericyte sites, and a two-tailed unpaired *t*-test was used for comparisons between SD and C57. For comparison between the immune components a nested one-way ANOVA and *post hoc* Tukey’s test were used. ^#^ *p* < 0.05 between component values; ***p < 0.001 for all other comparisons. Data from male SD and C57 show as black lines and error bars show means ± SEM; *n* ≥ 3 animals.

Video images of live tissue were collected to determine the effect of the inflammatory mediators on vessel diameter. Live tissue DIC imaging experiments were performed using a method previously described (Crawford et al., 2012). In brief, kidney slices were visualised on an unpright Olympus microscope (model BX51W1; Olympus microscopy) through a ×60 water immersion objective (0.9 NA; Olympus microscopy) where vasa recta were identified by their previously established “bump-on-a-log” morphology [Figure 1A (Peppiatt et al., 2006; Crawford et al., 2012)]. Subsequently, kidney tissue was superfused with PSS alone for approximately 100s (baseline), followed by PSS containing an inflammatory mediator for approximately 500s, and then subsequently subjected to a PSS wash out period. Inflammatory mediators chosen were those that have previously been shown to be upregulated and correlate with severity in human renal diseases (Siew et al., 2010; Gupta et al., 2012; Peng et al., 2012; Amdur et al., 2016; Gungor et al., 2017; Mende et al., 2018; Schröppel et al., 2019).The concentration of immune components used were informed by data available for their endogenous concentrations in renal disease (Lonnemann et al., 1999; Klos et al., 2013; Formanowicz et al., 2015; Gungor et al., 2017; Mende et al., 2018), but were often higher than pathophysiological concentrations since the superfusate in which they are contained needed to penetrate tissue to reach vasa recta capillaries ∼50 μm below the surface of the tissue slice. Figures 1Ai, Aii illustrate a typical field of view, including all medullary structures. Inflammatory mediators used for experimentation in the rat were TNF-α, IL-33, IL-1β, IL-18, C5a, and C3a, all at a concentration of 10 ng/mL (R&D Systems). In the mouse only TNF-α, IL-1β, and C5a were used at the same concentration and for the same duration. Live kidney slices for both rats and mice were only used for one experiment per slice, and only one vasa recta per kidney slice was used to ensure all vessels were “naïve” prior to exposure to any inflammatory mediator. As there is evidence for inhibition of the AT_1_ receptor improving outcomes following renal injury (Molinas et al., 2009; Gabriele et al., 2017), and the AT_1_-R role in pressor responses in the kidney (Ruan et al., 1997; 1999), and renal inflammation (Liu et al., 2012), experiments were conducted to asses its role in cytokine-elicited dysfunction. In experiments where rat tissue was co-exposed to AT_1_ receptor antagonist Losartan [100 nM, concentration determined previously as inhibitory of Ang-II 100 nM (Crawford et al., 2012); Tocris] in the presence of an inflammatory mediator, either inflammatory mediator or losartan were superfused across tissue slices alone for approximately 500s prior to tissue being superfused with both losartan and inflammatory mediator for approximately 500s, followed by a PSS “washout” period. Time-series analysis of kidney slice experiments was carried out off-line using the public domain software ImageJ (NIH, http://rsb.info.nih.gov.ij), as previously described (Crawford et al., 2012).

### Anti-NG2 immunohistochemistry and image analysis

Immunohistochemical experiments with kidney slices were used to investigate the effect of pro-inflammatory mediators on renal pericyte morphology and density. These were performed as previously described (Crawford et al., 2012). Live tissue was exposed to inflammatory mediators for 4-h prior to fixation and initiation of the immunohistochemistry protocol. Alexa fluor 488-conjugated isolectin B_4_ (IB_4_; 1:10 dilution in PSS, I21411, Invitrogen Ltd.) was used to identify the vasa recta capillaries in live tissue. Following fixation in 4% PFA in 0.1 M PBS and blocking in PBS containing 10% Donkey serum (Sigma-Aldrich Ltd.) and 0.1% Triton X-100 (10% DS/T; Sigma-Aldrich Ltd.). An anti-neural-glial 2 antibody (NG2; 1:200 dilution in DS/T; AB5320, Merck-Millipore) was used as a marker of pericytes. Anti-NG2 primary antibody was probed with donkey anti-rabbit Alexa 555 (A-21208; 1:200 dilution in DS/T; Invitrogen Ltd.) secondary antibody. Tissue slices were imaged using a ×63 oil immersion objective in a Nikon eclipse 50i microscope linked to a QICAM FAST 1394 digital camera. Alexa-Fluor 488 was excited at 488 nm and light collected with a band pass filter 505–515 nm, and Alexa-Fluor 555 was excited at 543 nm and light collected with a band pass filter 550–580 nm. Images were analysed off-line using ImageJ (NIH, http://rsb.info.nih.gov.ij) to measure pericyte density per 100 μm^2^, as well as pericyte soma morphology (soma height and width, and process length) and the corresponding vasa recta (vasa recta upon which the measured pericyte resides) diameter. To account for the sterology of the tissue sections (Bertram, 2001; Weibel et al., 2007), at least 14 and no more than 30 measurements were taken per treatment group.

### Statistical analysis

For analysis of the effect of inflammatory mediators (*treatment*) on pericyte density (*density*)*,* R packages *lme4* (https://cran.r-project.org/web/packages/lme4/index.html) and *emmeans* (https://cran.rstudio.com/web/packages/emmeans/) were used. Given the overdispersed nature of our count data, a negative binomial regression was used, with the model having the form *density ∼ treatment +* (*1* | *animal*). Assigning animal as a random effect was to account for the technical replicates in the data and within animal variation. We checked for violations of model fit for this model using a QQ-plot from the *DHARMa package* (https://cran.rstudio.com/web/packages/DHARMa/index.html) and a fitted versus Pearson residuals plot.

Graphpad PRISM 9.0 was used for statistical analysis of all normally distributed data sets (26, 27). DIC Statistics are calculated with the animal n, not the slice n. More than 1 slice per animal can be used as we previously found variation is between slices, not animals (Crawford et al., 2012). Statistical significance was calculated using the number of animals, with values from the same animal counted as technical replicates. For immunohistochemical statistics, n represents animals not number of measurements where again multiple measurements were treated as technical replicates. For DIC experiments, and murine immunohistochemical experiments, statistical significance was calculated using a nested two-tailed Student’s *t*-test that was paired or unpaired when relevant; *p* < 0.05 was considered significant. Regarding all other experiments, statistical significance was calculated using a nested one-way ANOVA and *post hoc* Dunnett test; *p* < 0.05 was considered significant. All values presented are expressed as mean ± SEM; number of animals and pericytes assessed per slice are presented as [*n* = animals(slices)], where only one experiment is conducted per slice.

## Results

### Inflammatory mediators evoke pericyte-mediated changes in *in situ* vasa recta capillary diameter

Figure 1A shows a typical field of view of a vasa recta capillary (DVR; Ai-ii) and corresponding temporal response profile (Aiii) to an agonist. To determine the acute effects of exposure of rat medullary DVR capillaries to key innate immune components, live kidney slices were superfused with C5a, IL-33, TNF-α and IL-18 (10 ng/mL); which resulted in a pericyte-mediated constriction of vasa recta capillaries. In all cases constriction of capillaries was significantly greater at pericyte sites (15.6% ± 1.1%, 9.2% ± 0.8%, 11.7% ± 0.8%, 9.8% ± 0.7%, respectively; Figure 1B) than at non-pericyte sites [1.0% ± 0.5%; *n* = 14(17); 1.9% ± 0.6%; *n* = 8(9); 0.6% ± 0.3%; *n* = 9(10); 0.7% ± 0.5%; *n* = 4(7); respectively; ***p < 0.001, Figure 1B]. The C5a-, IL-33-, TNF-α- and IL-18-mediated constriction of DVR was reversible at pericyte sites in 52%, 37%, 38% and 47% of experiments performed respectively. Exposure of live tissue to C3a (10 ng/mL) and IL-1β (10 ng/mL) failed to elicit a significantly greater change in vasa recta capillary diameter at pericyte sites (3.4% ± 0.5%, 2.2% ± 1.1%; data not shown) compared to non-pericyte sites [2.0% ± 0.8%; *n* = 4(6); 0.6% ± 0.2%, *n* = 10(19); *p* > 0.05; data not shown].

The C57BL/6J mouse is known to be relatively resistant to vasoconstrictors compared to the SD rat (Russell and Watts, 2000). Given that C5a and TNF-α elicited constrictions of greater magnitude in rat tissue than that elicited by IL-18 or IL-33, mouse tissue was subsequently only exposed to C5a and TNF-α with IL-1β as a non-responsive control. Both C5a and TNF-α elicited a pericyte-mediated constriction of vasa recta that was significantly greater at pericyte sites (7.4% ± 1.1% and 8.6% ± 0.6% respectively) than non-pericyte sites [2.7 ± 1.0; *n* = 5(5); and 2.5% ± 1.2%; *n* = 3(3); *p* < 0.05]. IL-1β failed to elicit a significantly greater change in capillary diameter (4.9% ± 1.6%) at pericyte sites compared to non-pericyte sites [2.8 ± 0.9; *n* = 4(4); *p* > 0.05]. Interestingly, the temporal aspect of the contractile responses to C5a and TNF-α appeared to be different in the two species (Figures 1Aii, Cii for exemplary C5a traces in rats and mice respectively). Murine contractile responses to C5a were of significantly lower magnitude than those recorded in the rat (Figure 1Ci, *p* = 0.0007), whilst TNF-α and iL-1β were not significantly different (Figure 1Cii). Only the rat slice model was used to further investigate if cytokine-evoked vasoconstriction involves angiotensin II type 1 receptors (AT_1_-R), as mouse vasa recta diameter did not return to baseline following removal of either C5a or TNF-α within the experimental protocol timeframe.

### Acute innate immune component-mediated vasoconstriction is AT_1_ receptor dependent

**FIGURE 2:**
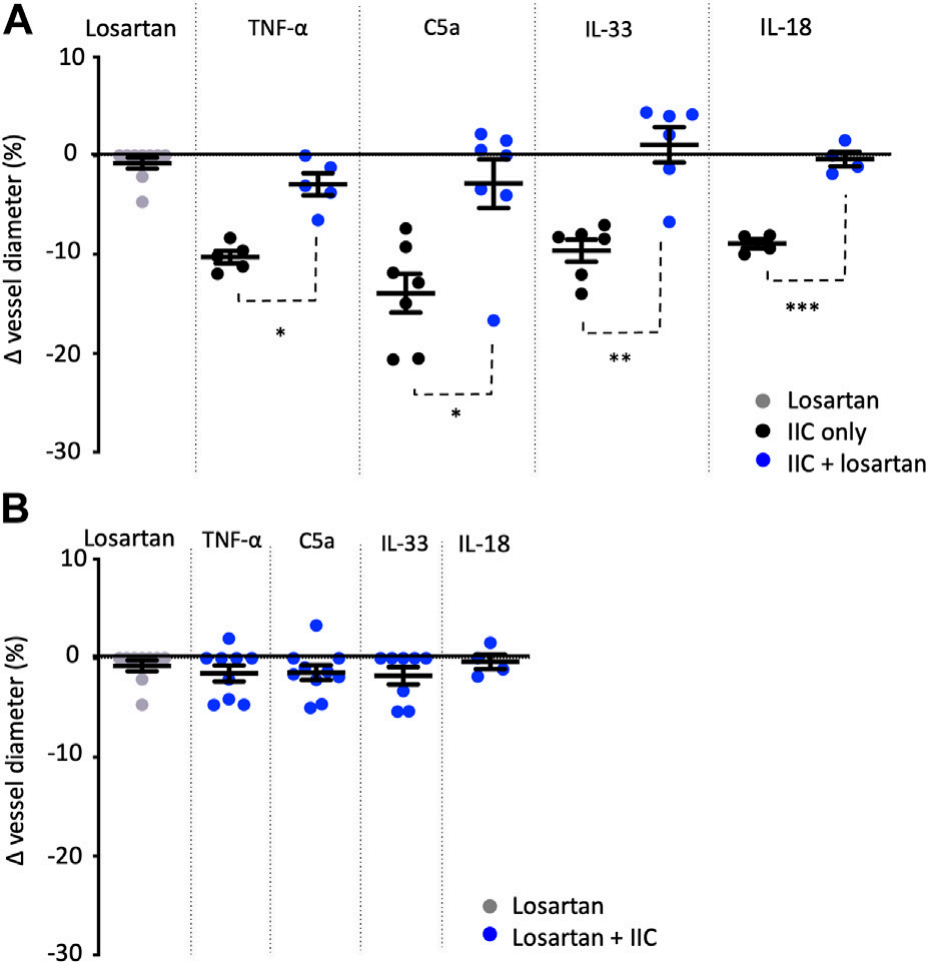
Losartan attenuates TNF-α-, C5a-, IL-33-, and IL-18-mediated constriction of rat vasa recta. Graphs show percentage changes in rat vasa recta diameter at sites in response to superfusion of tissue with losartan alone (grey spheres), innate immune components alone (IIC only; black spheres), and innate immune components combined with the AT_1_ antagonist losartan (blue spheres). Superfusion of tissue with losartan alone had no significant effect on vessel diameter at pericyte or non-pericyte sites. When tissue was superfused with TNF-α, C5a, IL-33, and IL-18 alone, a significant constriction of vasa recta was measured at pericyte sites compared to non-pericyte sites. The addition of losartan significantly attenuated the pericyte mediated constriction evoked by TNF-α, C5a, IL-33 and IL-18. No significant change in vessel diameter was observed at non-pericyte sites. **(A)** shows experiments where tissue was initially exposed to IIC alone and then in combination with losartan, and **(B)** shows experiments where tissue was exposed initially to losartan and then losartan in combination with IIC. Significance was calculated using a nested two-tailed unpaired Student’s *t*-test. **p < 0.01, ***p < 0.001. Data shown from male SD shown as means ± SEM; *n* ≥ 3 animals.

To determine whether the vasoconstriction evoked by C5a, IL-33, IL-18, and TNF-α involved AT_1_-R’s, live kidney slices were superfused with the innate immune components alone and in the presence of the AT_1_ receptor antagonist losartan (100 nM). Application of C5a, IL-33, IL-18 and TNF-α alone prior to inclusion of losartan in the superfusate induced a pericyte-mediated constriction (14.2% ± 0.9%, 9.7% ± 1.1%, 8.9% ± 0.6%, 10.7% ± 0.9%; Figure 2A) of vasa recta. The inclusion of losartan in the superfusate with each immune component, on average attenuated the pericyte-mediated constriction of vasa recta in response to C5a, IL-33, IL-18 and TNF-α by 78.6%, 111.6%, 96.6% and 65.6% respectively [*n* = 6(7), 6(6), 4(3), and 3(5) respectively; *p* < 0.05, Figure 2A]. No significant changes in vessel diameter were observed at non-pericyte sites throughout any of these experiments (data not shown).

To determine whether endogenous Ang-II activity may be contributing to pericyte-mediated vasoconstriction independently of C5a, IL-33 and TNF-α, kidney slices were superfused with losartan alone and then co-applied with immune components. Losartan alone caused no significant change in vessel diameter at pericyte sites (Figures 2A, B) compared to non-pericyte sites (*p* > 0.05; data not shown). Subsequent inclusion of innate immune components, C5a, IL-33, IL-18, and TNF-α in the superfusate with losartan, failed to elicit and further change in vessel diameter at pericyte (*p* > 0.05; Figure 2B) or non-pericyte sites (*p* > 0.05; data not shown).

### Prolonged exposure of kidney tissue to inflammatory mediators alters pericyte density, morphology and vessel diameter

**FIGURE 3:**
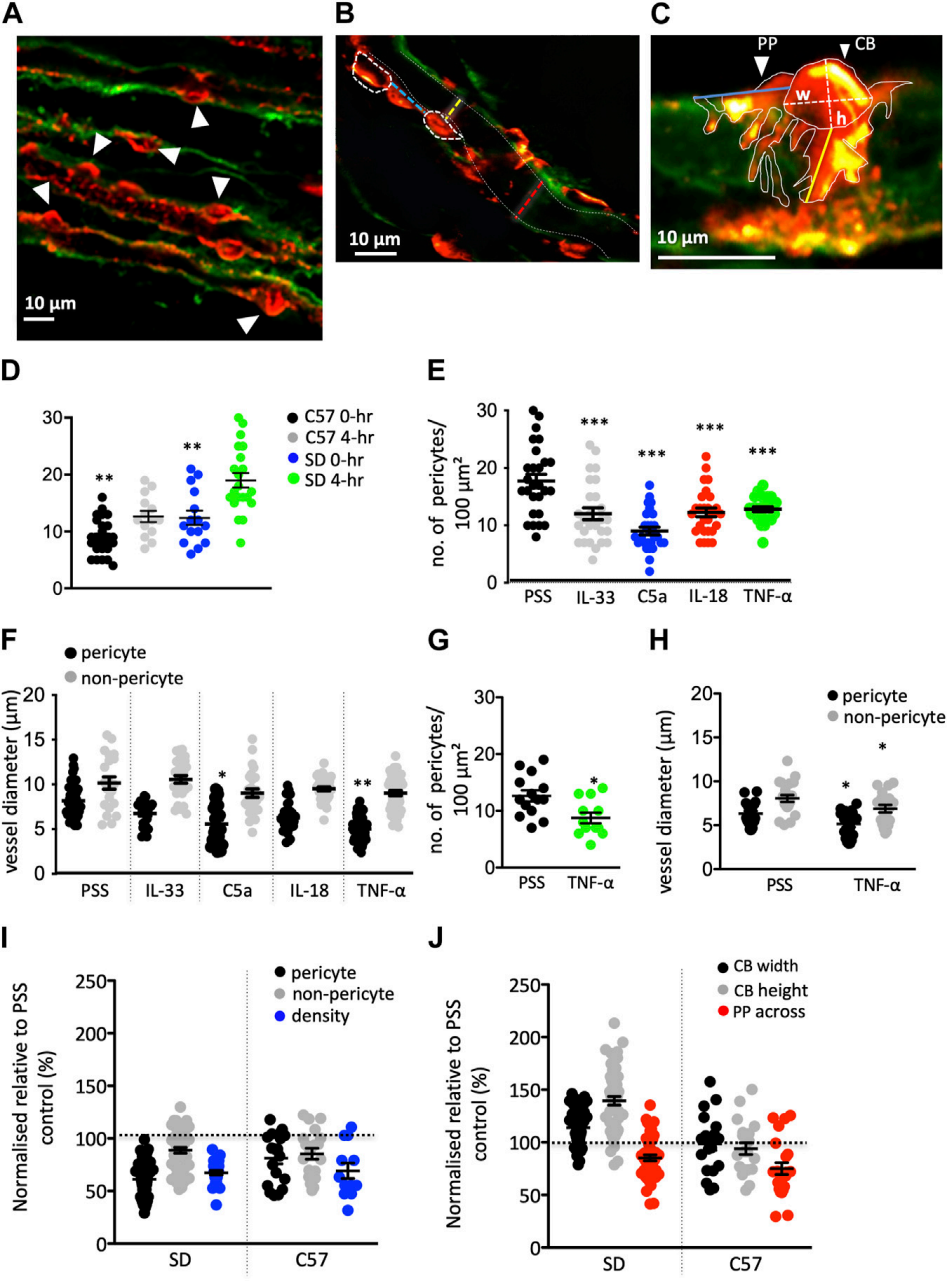
Innate immune components (IIC) changes in pericyte density, physiology, and vessel diameter in rat and murine tissue. **(A–C)** Images show typical fields of view of Sprague-Dawley (SD) rat vasculature labeled with IB_4_ (green) and pericytes labeled with anti-NG2 (red). **(A)** Image shows pericyte density (white arrowheads indicating pericytes) in an area of 100 μm^2^. **(B)** shows pericyte distribution along an individual vasa recta [white dashed ovals outline pericyte cell bodies (CB)] with the distance between indicated by the blue dashed line. Yellow and red dashed lines indicate where vasa recta diameter was measured at a pericyte and non-pericyte site respectively. White dashed lines indicate the capillary walls. **(C)** Image shows pericyte CB and pericyte processes (PP) outlined with solid white lines. Where the CB height (h) and width (w) measurements taken are denoted by dashed white lines. Longitudinal (PP along) and circumferential (PP across) process measurements for individual pericyte soma were also taken, indicated by the blue and yellow lines respectively. Graph **(D)** illustrates a significant difference in the density of pericytes after 4 h of incubation under control PSS conditions in both rats and C57BL/6J mice (C57). Graphs **(E,F)** show the number of pericytes within an area of 100 μm^2^ **(E)**, vessel diameter at pericyte and non-pericyte sites **(F)** after exposure to IIC in SD tissue. All stimuli evoked a significant decrease in pericyte density in an area of 100 μm^2^, compared to PSS time matched control group. Vasa recta diameter was also significantly reduced at pericyte-sites for C5a and TNF-α with no significant change measured at non-pericyte sites. Graphs **(G,H)** show the number of pericytes within an area of 100 μm^2^ **(G)**, vessel diameter at pericyte and non-pericyte sites **(H)** after exposure to TNF- α in C57 tissue. **(I)** Graph shows PSS-normalised values for vasa recta diameter at pericyte and non-pericyte sites, and pericyte density SD rats and C57 upon exposure to TNF-α, with **(J)** showing PSS-normalised data for all other measurements taken (CB width and height, and PP across). The PSS average for each metric was set as “100%” (black dashed line). What can be seen are a consistent reduction in pericyte density and pericyte site diameter after TNF-α stimulation, with changes in CB morphology unique to the rat. Data in [**(D,E,G)]** are count data and as such significance was calculated using a negative binomial regression with the form *Density ∼ treatment +* (*1* | *animal*) for both rats and mice. Significance for all other SD data here was calculated using a nested one-way ANOVA with *post hoc* Dunnet’s test with PSS data as the control. A nested two-tailed paired Student’s *t*-test was used to calculate significance in the C57 for all other data. All statistics shown are for treated pericytes vs. PSS control pericytes within rats or mice. *p < 0.05, **p < 0.01, ***p < 0.001. Data shown from male SD and C57 shown as black lines and error bars show means ± SEM; *n* = 3 animals in the SD and C57.

Having investigated the effect of acute exposure of kidney tissue to a series of innate immune components, we sought to further investigate the impact of exposing live kidney tissue to the same components for a period of 4 h. We have established previously that our kidney slices are viable for 4-h (Crawford et al., 2012) whilst other labs have found tissue is viable for up to 72-h (Poosti et al., 2015; Stribos et al., 2016). Following fixation of this tissue and fluorescence imaging of immunohistochemically labelled percytes and vasa recta capillaries, we measured the denisity of NG2^+^ pericyte cells along vasa recta capillaries (in 100 μm^2^ of tissue), pericyte process length (around vasa recta), pericyte cell body length and width, and vessel diameter (at pericyte site and non-pericyte sites). Fluorescence images of fixed tissue slices exposed to immune components and PSS alone were acquired (Figures 3A–C) and analysed off-line.

Exposure of rat tissue to IL-33, C5a, IL-18, and TNF-α (4 h, compared to 4 h treatment with PSS alone), prior to fixation, resulted in a significant decrease in pericyte density (*p* < 0.01 Figure 3D) and vessel diameter at pericyte sites for C5a and TNF-α (*p* < 0.05 and *p* < 0.01 respectively; Figure 3E) compared to those measured in tissue exposed to PSS only (see Table 1 for values, *n* = 3). There was no significant difference in vessel diameter (Figure 3E) at non-pericyte sites in response to innate immune components compared to PSS control experiments [Table 1, *n* = 3(3)]. There was no significant change in pericyte cell body width in response to IL-33, IL-18, TNF-α or C5a compared to PSS control experiments {Table 1, *n* = 3(3), yet there was a significant increase in pericyte cell body height [*p* < 0.01; Table 1, *n* = 3(3)]}.

**TABLE 1:** Summary table of anti-NG2 immunohistochemistry experiment data. Table shows the measurements for pericyte density per 100 μm^2^ (Pericyte no.), pericyte morphology, and vessel diameter in slices exposed to innate immune components and PSS and corresponding significance values (PSS vs. innate immune component).

Innate immune component	Pericyte no.	Cell body width (μm)	Cell body height (μm)	Process length across vessel (μm)	Vessel diameter at pericyte sites (μm)	Vessel diameter at non-pericyte sites (μm)	*Fold increase* vs*. acute superfusion*
	Sprague-Dawley rat						
PSS	19.0 ± 1.3	9.3 ± 0.3	3.5 ± 0.2	8.6 ± 0.4	8.4 ± 0.3	10.8 ± 0.8	N/A
C5a	9.0 ± 0.7	7.6 ± 0.3	5.2 ± 0.2	5.8 ± 0.5	5.6 ± 0.4	9.0 ± 0.5	*(15.2%* vs*. 32%) approximately double*
	***p < 0.001	*p* > 0.05	**p < 0.01	*p < 0.05	*p < 0.05	*p* > 0.05	
IL-33	12.0 ± 1.0	8.5 ± 0.3	4.8 ± 0.1	5.8 ± 0.4	6.7 ± 0.2	10.6 ± 0.3	*(9.21%* vs*. 19%) approximately double*
	***p < 0.001	*p* > 0.05	**p < 0.01	*p < 0.05	*p* > 0.05	*p* > 0.05	
TNF-α	12.8 ± 0.4	8.6 ± 0.2	5.1 ± 0.1	7.3 ± 0.2	5.1 ± 0.2	9.0 ± 0.3	*(11.7%* vs*. 39%) approximately 4-fold*
	***p < 0.001	*p* > 0.05	**p < 0.01	*p* > 0.05	**p < 0.01	*p* > 0.05	
IL-18	12.3 ± 0.8	8.6 ± 0.2	4.9 ± 0.2	6.9 ± 0.3	6.2 ± 0.3	9.5 ± 0.2	*(9.8%* vs*. 23%) approximately 2.5-fold*
	***p < 0.001	*p* > 0.05	**p < 0.01	*p* > 0.05	*p* > 0.05	*p* > 0.05	
	C57BL/6J Mouse						
PSS	12.6 ± 1.0	7.5 ± 0.4	4.5 ± 0.2	6.8 ± 0.5	6.3 ± 0.3	8.1 ± 0.4	N/A
TNF-α	8.8 ± 0.9	7.4 ± 0.5	4.3 ± 0.3	5.1 ± 0.4	5.1 ± 0.3	6.9 ± 0.4	(*8.6%* vs*. 17%) approximately double*
	*p < 0.05	*p* > 0.05	*p* > 0.05	*p < 0.05	*p < 0.05	*p < 0.05	

Values are means ± SEM; *n* = 3 animals. A nested two-tailed paired Student’s *t*-test was used to calculate significance in the male C57BL/6J mouse. Significance in the male Sprague-Dawley rat was calculated using a nested one-way ANOVA and *post hoc* Dunnet test. *p < 0.05, **p < 0.01, ***p < 0.001 cytokines vs. PSS.

Given that there was no significant difference in the TNF-α mediated constriction of vasa recta in mouse tissue when compared to that in rat (unlike C5a), mouse tissue was exposed to TNF-α only in this set of experiments. In murine tissue, the basal pericyte density was lower than in rats, and the vasa recta capillaries are narrower (Table 1; Figures 3F, I). Exposure of murine tissue to TNF-α, prior to fixation, resulted in a significantly greater decrease in pericyte density (*p* < 0.05; Figure 3F), vasa recta diameter (*p* < 0.05; Figure 3G) and circumferential process length (*p* < 0.05; Table 1) compred to that measured in mouse tissue exposed to PSS alone [Table 1, *n* = 3(3) for TNF-α and 4(4) for PSS]. No significant changes in pericyte cell body width or height were detected in tissue treated with TNF-α when compared to tissue treated with PSS alone (Table 1).

Given the absolute values of measurements were different between rats and mice, data was normalized (using PSS control averages) for comparison between the species of the relative magnitude of change after exposure to TNF-α. When comparing the normalized TNF-α evoked changes in mouse and rat tissue, it was noted that the relative change in pericyte density (Figure 3H) vasa recta diameter (Figure 3H), and circumferential process length (Figure 3I) after exposure to TNF-α were not significantly different between species, whilst the changes in pericyte soma morphology showed differences between the species (Figure 3J).

## Discussion

In the present study we have demonstrated that 1) innate immune components can elicit a pericyte-mediated constriction of vasa recta, 2) they elicit this acute vasoconstriction, in part, via activity at Ang-II receptor type 1 (AT_1_-R), and 3) sustained exposure to inflammatory mediators led to a reduction in number, a contraction of pericyte processes, and a sustained vasoconstriction of NG2^+^ pericytes**.** Taken together, whilst the dysregulation of pericyte-mediated regulation of vasa recta diameter is not the sole pathophysiological process following renal injury, we have shown inflammatory mediators act at renal pericytes in the medulla acutely to dysregulate vasa recta diameter and thus are potentially involved in the decline in MBF and may induce pathological cellular events that are associated with AKI.

Circulating immune cells release pro-inflammatory mediators as they bind to the endothelium (Bonventre and Yang, 2011). Several studies, covering a range of species and vascular beds, have shown inflammatory mediators can be directly and indirectly vasoactive (Vila and Salaices, 2005; Shahid et al., 2008; Zhang et al., 2014b). Direct vasoactivity involves action at cytokine receptors on endothelial, vascular smooth muscle cells (vSMC) (Baudry et al., 1996; Vila and Salaices, 2005), and pericytes (Kerkar et al., 2006). Indirect vasoactivity involves the downstream release of vasodilatory mediators like NO and PGI_2_ by upregulating COX-2 and iNOS, as well as stimulating endothelial production of vasoconstrictive mediators including ROS, Ang-II and ET-1 (Vila and Salaices, 2005; Shahid et al., 2008; Zhang et al., 2014b). Unfortunately, whilst a comparable data set does noy exist for the rat, murine renal pericytes have had their genes profiled related to those of other medullary cells and it was found they express more interleukin-1 receptor (IL-1R)-II, IL-1R-like 2, and TNF-receptor (-R) superfamily members 1b, 9, 11, and 11b relative to other medullary cells, whilst the surrounding medulla expresses more complement receptors, IL-1R receptor-like 1, IL-1RI (Grgic et al., 2014). As such data supports direct activity of inflammatory mediators at renal pericytes. If rat renal pericytes were to express more complement receptors and comparable TNF-R it could partly explain differences in the responsiveness between species. However, we believe the vasoconstrictions observed to be the net result of the stimulation with cytokines and the activation of all associated downstream signalling pathways. With the live slice model, these signalling pathways are preserved and the intravascular, extravascular, and tubular spaces exposed to the inflammatory mediator concurrently. As adjacent tubules have shown to influence the vasoactivity of pericytes (Cowley and O’connor, 2013), as well as endothelial-pericyte communications (Armulik et al., 2011) a mediator does not need to act directly at pericytes to elicit vasoconstriction. Our imaging of live rat and murine kidney tissue enabled us to show in real-time that the net result of acute exposure to IRI-associated complement proteins (C5a) and cytokines (IL-33, IL-18 and TNF- α) is a pericyte-mediated constriction of vasa recta. Further still, our study demonstrates that this pericyte-mediated constriction involves activity at the Ang-II type 1 receptor (AT_1_-R) in the rat, and thus Ang-II. Whilst not the most significant pathway involved in IRI-induced changes to MBF, the activation of AT_1_-R has been implicated in no-reflow following renal IRI (Freitas and Attwell, 2022), and AT_1_-R blocker losartan impairs post-ischaemic cytokine production and limits injury (Molinas et al., 2009; Gabriele et al., 2017). In experiements shown here, superfusion with losartan alone elicited no change in vessel diameter, whilst superfusion of losartan together with innate immune component significantly attenuated the immune component mediated vasoconstriction. This would suggest inflammatory mediators stimulate renal cells to produce Ang-II that is not present in un-stimulated tissue. This data agrees with other studies where C3a induces Ang-II production in vSMC from spontaneously hypertensive rats (SHR) (Han et al., 2012); and both TNF-α (Zhang et al., 2014b) and C5a (Zhang et al., 2014a) are linked with Ang-II-induced hypertension, CKD, and cardiac injury in murine models.

The vasoctivity of innate immune components involving AT_1_-R could explain vascular-bed specific differences in cytokine behaviour, and the species difference in magnitude of pericyte-mediated constriction (Figure 1Cii). Upon exposure to TNF-α, a vasodilation is measured in the rat cremaster muscle (Baudry et al., 1996) whilst it constricts the rat and murine renal vasculature (Shahid et al., 2008; Zhang et al., 2014b). If we relate this vasoactivity to AT-R distribution, the rat cremaster muscle expresses more dilatory AT_2_-R (Hong et al., 2016) than rat kidneys (Ruan et al., 1997), and offers an explanation for the observed differences. An AT-R dependent difference in Ang-II contractility has also been shown in the mesentery of aged mice where reduction in AT_2_-R approximately doubles the contractility of Ang-II comparative to young mice (Dinh et al., 2017). Moreover, the SD rat kidney has approximately double the AT_1_-R density of the C57BL/6J mouse (Cassis et al., 2004), and thus a greater magnitude of pericyte-mediated contractility. Given the fundamental role of the renin-angiotensin system in kidney function it is perhaps unsurprising that there might be a role for AT_1_-R in the immune-component elicited pericyte-mediated constriction of DVR. However, only performing losartan experiments in rat tissue means these finding may not generalise as described here, and not repeating these experiments in mice is a noted limitation of this study.

Conflicting somewhat with other work highlighting roles for C3a and IL-1β in the pathogenesis of IRI-AKI (Rusai et al., 2008; Peng et al., 2012) these immune components failed to elicit acute pericyte-mediated changes in DVR diameter, yet there are other ways they may contribute to heamodynamic dysregulation. In the SHR, IL-1β potentiates the vasoactive response of phenylephrine, ET-1, and Ang-II in the mesentery, cremaster and heart (Baudry et al., 1996; Vicaut et al., 1996; De Salvatore et al., 2003; Dorrance, 2007) opposed to direct vasoactivity. Our acute experimental window may also be insufficient to induce indirect vasomotor activity; the cytokine upregulation induced by C5a and C3a is time-dependent (Monsinjon et al., 2003). Another consideration is non-vasoactive actions that cause injury and may lead to impaired MBF. Antagonism of IL-1 receptors has been shown to alter lymphocyte and macrophage infiltration, and IL-1α/β knock out mice exhibit reduced acute tubular necrosis between 24 and 48 h after ischemia (Rusai et al., 2008). This immune cell infiltrate occludes vasa recta (Kelly et al., 1996; Naruse et al., 2002; Ysebaert et al., 2004; Kielar et al., 2005; Basile et al., 2012) and could impede blood flow independently of the actions of pericytes. So, whilst C3a and IL-1β contribute to kidney injury (Rusai et al., 2008; Peng et al., 2012), these mechanisms appear independent from acute pericyte-mediated MBF dysregulation and are beyond the scope of this study.

In longer term experiments, where tissue was exposed to innate immune components for 4 h, vessels remain strangulated by pericytes. In these incubations vessel constriction was not only sustained but approximately double that of the acute experiments. Previously, we, and others, have argued that pericyte-mediated changes in DVR diameter are likely to underpin localized changes in blood flow, and dysregulation of MBF via pericyte cells is likely to underpin many renal pathologies (Kennedy-Lydon et al., 2013; 2015; Peppiatt-Wildman, 2013; Lemos et al., 2016; Xavier et al., 2017). Whilst we cannot say for certain constriction was sustained from the start of incubation, 4 h of reduced blood flow, due to pericyte strangulation, is a substantial amount of time for tissue to be under perfused and likely to cause ischaemic and hypoxic conditions. Recently it has been shown that NG2^+^ pericytes mediate the medullary no-reflow in renal ischaemia (Freitas and Attwell, 2022), we further propose that the renal injury-associated innate immune components tested here might also play a role in the sustained dysregulation of MBF that occurs during ischemia-reperfusion.

As mentioned in the introduction, a sustained dysregulation of MBF is a notable feature of ischaemic AKI (Regner and Roman, 2012), and whilst our work here, and that of others (Crislip et al., 2017; Freitas and Attwell, 2022) highlight a pericyte-mediated role for this reduction there exists further factors involved in the dysfunction of outer medullary perfusion. There are other structural changes that cause the noted capillary narrowing and occlusion in AKI (Ray et al., 2019). Medullary congestion by infiltrating leukocytes is an early notable feature of kidney injury (De Greef et al., 2001; de Greef et al., 2003; Ysebaert et al., 2004; Kielar et al., 2005; Basile et al., 2012; Crislip et al., 2017), and severe endothelial and tubular swelling (Mason et al., 1984) increases pressure on the medullary capillaries causing occlusion. Inflammatory cells also have high O_2_ requirements (Murdoch et al., 2005), however the renal medulla normally functions in a borderline hypoxic state (Ray et al., 2019). The highly metabolically active S3 segment of the proximal tubule in the outr medulla, with a limited capacity for anaerobic function (Guzzi et al., 2019), and the medullary thick ascending limb are also highly vulnerable to hypoxic insult and renal injury, with tubular dilation and loss of microvilli noted within hours of reperfusion (Basile et al., 2012). This tubular hypoxia results in ATP depletion (Basile et al., 2012), and mitochondrial swelling and dysfunction promoting apoptotic cytochrome C release and cell death (Legrand et al., 2008; Duann and Lin, 2017). The impaired energetics from hypoxic tubules also causes a loss of function, tubular cells lose their brush border and the breakdown of the cytoskeletal structure leads to a loss of epithelial polarity and function (Basile et al., 2012). This dyregulation itself could elicit a secondary reduction in MBF from the reduction in tubular sodium handling (Fan et al., 2019). Unfortunately, our study was focused on the potential contribution of pericytes to the medullary vascular dysfunction and as such we did not probe and tubular dysfunction here and as such we cannot comment on their contributions to the dysregulation we noted following inflammatory mediator exposure.

We have also shown that stimulation with innate immune components results in a decrease in pericyte density across the medulla comparative to PSS control experiments in both rats and mice. In the SHR, loss of NG2^+^ pericytes is associated with increased injury following renal ischaemia (Crislip et al., 2017), with a recent porcine IRI model demonstrating this dysregulation of the medullary vasculature elicited by inflammation; inhibiting C5a ameliorates the significant reduction in NG2^+^ pericyte number and capillary constriction at 24 h post-reperfusion (Castellano et al., 2018). Our work further complements these studies showing other IRI-associated inflammatory mediators cause this vascular dysregulation, and how rapidly this can occur. However, a limitation of our work here is we did not further probe what happened to these pericytes and we cannot conclusively comment on the subsequent fate of these NG2^+^ pericytes.

Following calculating the pericyte densities we also sought to quantify changes in pericyte morphology in response to innate immune components. We have demonstrated that inflammatory mediators induce contractions of circumferential processes, but the morphological change of pericyte soma is only in rats. Interestingly, this would suggest that whilst the constriction of vasa recta diameter is conserved, morphological changes are unlikely to be generalizable findings. The reduction in pericyte density and retraction of pericyte processes is supported by a previous study where injection of TNF-a and IL-1β induced a loose and non-confluent coverage of pericytes along postcapillary venules in murine cremaster muscle and ear skin (Proebstl et al., 2012). Whilst this relaxation of pericyte processes is necessary for leukocyte extravasation (Wang et al., 2012), this process could initiate a pericyte-myofibroblast transition as this pericyte relaxation (Wang et al., 2012), leukocyte migration (Cernuda-Morollón and Ridley, 2006), and fibrotic development (Baba et al., 2015) are Rho-A/ROCK dependent, though further work is needed to elucidate this mechanism. Further to this, Freitas and Attwell (Freitas and Attwell, 2022) demonstrated how Rho-A/ROCK inihibition restores MBF fastest following IRI, and inihibits the no-reflow phenomenon specifically by activity at NG2^+^ pericytes. Pericytes provide stability to the capillaries on which they reside (Lemos et al., 2016), and their loss induces capillary rarefaction (Kramann et al., 2017) which culminates in a loss of up to 50% of the vasa recta capillaries in rats 4-weeks after ischaemia (Basile et al., 2001; Regner and Roman, 2012). Our data here, and others (Crislip et al., 2017; Castellano et al., 2018), demonstrating an early dysregulation of NG2^+^ pericytes is possibly linked to the vessel instability and rarefaction observed renal disease (Lemos et al., 2016; Kramann et al., 2017).

No method is without limitations, and whilst the live kidney slice model offers an *in situ* model that retains the 3-dimensional cellular complexity and cellular interactions/communications present *in vivo* (Bach et al., 1996; Vickers and Fisher, 2005; Crawford et al., 2012), the nature of this technique means that the corticomedullary concentration gradients are not preserved, and that the vasculature is not perfused and consequently we cannot explicitly determine with this model if the microvascular dysfunction we have demonstrated elicits a reduction of MBF. The high doses of cytokines used, whilst based upon *in vivo* concentrations disease (Lonnemann et al., 1999; Klos et al., 2013; Formanowicz et al., 2015; Gungor et al., 2017; Mende et al., 2018), may not reflect the dynamic temporal nature of cytokine production (Behrsing et al., 2013) as concentrations are kept constant in our superfusion and incubation experiments. Whilst we also demonstrate a cytokine elicited pericyte dysfunction across different species, we only use male animals and thus cannot comment on sex differences even though in rats some sex difference in ischaemic renal injury have been established (Crislip et al., 2017). However, despite these limitations our findings are consistent with the work of other groups who used *in vivo* experimentation to investigate pericyte dysregulation in renal injury (Crislip et al., 2017; Freitas and Attwell, 2022) and as such suggests the kidney slice model is beneficial for elucidating the cellular mechanisms in the otherwise inaccessible medulla.

## Conclusion

In conclusion, we have provided novel evidence demonstrating inflammatory mediators can evoke acute pericyte-mediated changes in vasa recta diameter, as well as changes in NG2^+^ pericyte density and morphology. These effects could underpin the sustained dysregulation of MBF in IRI, leading to hypoxic cellular and tissue damage and subsequent renal dysfunction. All of these mechanisms have been linked with AKI and the transition to CKD, and possibly underlie early cellular events that lead to pericyte detachment and differentiation into myofibroblasts. Whilst further work is needed, delineating these mechanisms could highlight potential therapeutic targets to prevent the onset of AKI following IRI*.*

## Data Availability

The raw data supporting the conclusion of this article will be made available by the authors, without undue reservation.
